# Cotinine versus questionnaire: early-life environmental tobacco smoke exposure and incident asthma

**DOI:** 10.1186/1471-2431-12-187

**Published:** 2012-12-05

**Authors:** Chris Carlsten, Helen Dimich-Ward, Anne DyBuncio, Allan B Becker, Moira Chan-Yeung

**Affiliations:** 1 Department of Medicine, University of British Columbia, Vancouver, BC, Canada; 2 Department of Pediatrics, University of Manitoba, Winnipeg, MB, Canada

**Keywords:** Children, Exposure to environmental tobacco smoke, Bronchial hyperresponsiveness, Wheeze, Asthma

## Abstract

**Background:**

The use of biomarkers has expanded considerably, as an alternative to questionnaire-based metrics of environmental tobacco smoke (ETS); few studies have assessed the affect of such alternative metrics on diverse respiratory outcomes in children, and we aimed to do so.

**Methods:**

We evaluated various measures of birth-year ETS, in association with multiple respiratory endpoints early years of life, in the novel context of a birth cohort at high risk for asthma. We administered questionnaires to parents, both at the end of pregnancy and at one year of life, and measured cotinine in cord blood (CCot; in 275 children) and in urine (UCot; obtained at 12 months in 365 children), each by radioimmunoassay. Multiple logistic regression was used to assess the association of the various metrics with recurrent wheeze at age 2 and with bronchial hyperresponsiveness (BHR) and asthma at age 7.

**Results:**

Self-reported 3rd trimester maternal smoking was associated with significantly increased risk for recurrent wheeze at age 2 (odds ratio 3.5 [95% confidence interval = 1.2,10.7]); the risks associated with CCot and 3rd trimester smoking in any family member were similar (OR 2.9 [1.2,7.0] and 2.6 [1.0,6.5], respectively). No metric of maternal smoking at 12 months appeared to significantly influence the risk of recurrent wheeze at age 2, and no metric of ETS at any time appeared to significantly influence risk of asthma or BHR at age 7.

**Conclusions:**

Biomarker- and questionnaire-based assessment of ETS in early life lead to similar estimates of ETS-associated risk of recurrent wheeze and asthma.

## Background

Exposures that confer risk for incident childhood asthma include early-life stressors and infections, allergens, outdoor pollutants, and tobacco smoke [1]. In our previous work [2], we focused on the lattermost and demonstrated that questionnaire-based exposure to environmental tobacco smoke (ETS) at year 7, administered to parents within a birth cohort of high-risk children, was not a risk factor for asthma at year 7. An alternative to questionnaire-based data is to examine cotinine as a direct metric of exposure. A recent comprehensive meta-analysis [3], which included studies of older children, converted cotinine measures to approximate cigarette dose as an alternative to comparing the effect of the various metrics on risk estimates directly. However, few studies have assessed the effect of multiple ETS exposure metrics on diverse respiratory outcomes over a number of years. We hypothesized that biomarker-based assessment of ETS and questionnaire-based assessment of ETS would lead to different estimates of ETS-associated asthma risk. The purpose of the paper is to compare questionnaire-based ETS estimates with biomarkers of ETS, in terms of associated risk for recurrent wheeze, physician-diagnosed asthma, and measures of bronchial hyperresponsiveness (BHR), using a unique birth cohort.

## Methods

The cohort consisted of 545 children from Vancouver and Winnipeg at high-risk of asthma, 380 (70%) of whom were followed over 7 years. High-risk was defined as having, according to parental report, at least one first-degree relative with asthma or two first-degree relatives with other IgE-mediated allergic disease (atopic dermatitis, seasonal or perennial allergic rhinitis, or food allergy). We successfully measured cotinine in cord blood (CCot; in ng/ml) obtained at birth in 275 children and urinary cotinine:creatine (UCot, in ng/mg) obtained at 12 months in 365 children, each by radioimmunoassay kits (Brandeis University, Department of Biochemistry, Waltham, MA). The limit of detection of cotinine is 200 pg/ml. As previously reported [4,5], questionnaire data were obtained in person (via parents) during the third trimester of pregnancy and at one, 2 and 7 years of age, and asthma and BHR were determined at age 7. Asthma was defined as at least 2 or more distinct episodes of cough, each lasting at least 2 or more weeks, at least 2 distinct episodes of wheeze, each lasting at least one or more weeks; plus at least 1 of the following: nocturnal cough (at least once a week) in the absence of a cold, hyperpnea-induced cough or wheeze at any time, or response to treatment with beta-agonist and/or anti-inflammatory drugs. Recurrent wheeze at age 2 was defined as at least 3 episodes of wheezing in the past 12 months reported by questionnaire. Logistic regression, with covariates as noted below, was used to determine the odds ratio for a given outcome associated with maternal smoking during the 3^rd^ trimester, any family member smoking during the 3^rd^ trimester, and measurement of cord blood cotinine at the time of birth, as well as maternal smoking, any family member smoking and urinary cotinine and creatinine, at one year of age, as detailed further in Table 1. Individual smoking was a dichotomized variable whereas cotinine measures were continuous variables. Only those children with data on each of the ETS metrics for a given exposure window were included. The fluxogram (Figure 1) summarizes the flow of our study.

**Table 1:** Risk of asthma, recurrent wheeze and bronchial hyperreactivity with various metrics of environmental tobacco smoke

		**2 years**			**7 years**					
		**Recurrent wheeze**			**Asthma**			**Bronchial hyperreactivity**		
		**Yes**	**No**	Adjusted* OR^**#**^	**Yes**	**No**	**Adjusted OR**	**Yes**	**No**	**Adjusted OR**
Measures at 3^rd^ trimester/birth	Maternal smoking (self-reported) during 3rd trimester	5/23 (21.7)	18/252 (7.1)	**3.5 (1.2,10.7) p=0.025**	4/50 (8.0)	19/229 (8.3)	0.5 (0.1,1.6)	11/146 (7.5)	9/112 (8.0)	0.9 (0.4,2.3)
	Any family member smoking (self-reported) during 3rd trimester	11/23 (47.8)	60/252 (23.8)	**2.9 (1.2,7.0) p=0.015**	14/50 (28.0)	58/229 (25.3)	0.9 (0.4,1.9)	38/146 (26.0)	29/112 (25.9)	1.0 (0.6,1.8)
	Cord blood cotinine above 50^th^ percentile	16/23 (69.6)	119/252 (47.2)	**2.6 (1.0,6.5) p=0.045**	31/50 (62.0)	106/229 (46.3)	1.7 (0.9,3.2)	74/146 (50.7)	51/112 (45.5)	1.2 (0.7,2.0)
Measures at 12 months	Maternal smoking (self-reported) at 12 months	4/33 (12.1)	29/332 (8.7)	1.3 (0.4,4.1)	5/71 (7.0)	28/299 (9.4)	0.3 (0.1,1.0)	15/188 (8.0)	15/150 (10.0)	0.8 (0.4,1.7)
	Any family member smoking (self-reported) at 12 months	10/33 (30.3)	66/332 (19.9)	1.7 (0.8,3.8)	15/71 (21.1)	62/299 (20.7)	0.7 (0.3,1.4)	45/188 (23.9)	27/150 (18.0)	1.5 (0.9,2.7)
	Urinary cotinine:Cr above 50th percentile at 12 months	23/33 (69.7)	167/332 (50.3)	2.2 (0.99,4.7)	43/71 (60.6)	149/299 (49.8)	1.2 (0.7,2.1)	94/188 (50.0)	79/150 (52.7)	0.9 (0.6,1.3)

**BOLD** indicates p < 0.05 frequency of exposure at given age amongst those with (“yes”) or without (“no”) given diagnosis.

* each model adjusted for group allocation (“intervention”) and for any baseline characteristic (race gender, history of asthma, maternal education, residence city, season, child’s atopic status) with p<0.05 upon being entered in a stepwise manner.

# odds of having given respiratory condition amongst those with given ETS exposure relative to those without such ETS exposure (95% CI).

**Figure 1:**
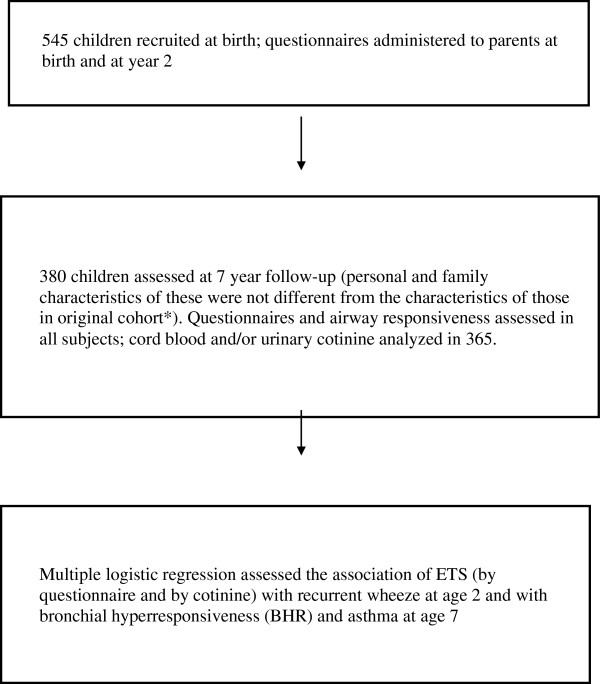
Flow chart of essential study features.

## Results

Reported maternal smoking at any timepoint assessed was infrequent (8% during the 3^rd^ trimester and 9% at one year of age). Amongst those mothers who smoked during the 3^rd^ trimester, the mean (± SD) number of cigarettes per day was 10 (± 7). 69% of those reduced smoking during pregnancy. The Pearson correlation (*r*) between a positive self-report of maternal smoking during the third trimester of pregnancy and CCot was 0.89 (p < 0.0001). CCot ranged from 0 (below the detection limit) to 330 ng/ml; UCot ranged from 0 (below the detection limit) to 2821 ng/mg. The *r* between a positive self-report of maternal smoking at 12 months and urinary cotinine was 0.48 (p < 0.0001). The correlations between any family member reporting smoking during 3^rd^ trimester or at 12 months, and CCot or UCot (respectively), were 0.52 (p < 0.0001) and 0.47 (p < 0.0001). The *r* between UCot and CCot was 0.54 (p < 0.0001).

As noted in Table 1, the choice of ETS metric during a given exposure time-frame influences the estimate of asthma risk only modestly; within a given exposure time-frame the various measures generally confer similar risk for a given respiratory outcome. For exposures near the time of birth, all 3 metrics were associated with significantly increased risk for recurrent wheeze at year 2, but none was associated with significant risk of wheeze or asthma of the year 7 outcomes. Regarding the measures determined at 12 months, there is a trend towards increased risk for recurrent wheeze at 2 years, with the objective measure of UCot being associated with a borderline significant risk for recurrent wheeze (OR 2.2, [0.99, 4.7], p = 0.051). Regarding asthma and BHR at 7 years, no metric was significantly associated with risk. There was a suggestion that self-reported metrics conferred relative “protection” for asthma at 7 years (borderline statistical significance) while the opposite trend was apparent based on the biomarkers. We did sensitivity analyses using alternate cutoffs for elevated CCOt and UCot, based on prior literature [6,7], but this did not appreciably change the results.

## Discussion

In the 1980’s large cohort studies showed that ETS exposure, assessed by parental questionnaire, was associated with increased risk for BHR in children [8]. Subsequently, investigators moved to gathering biomarkers of exposure from the children themselves. Children’s urinary cotinine was demonstrated as a biomarker of risk for asthma and wheeze [9]. Subsequently, the use of biomarkers for ETS has expanded considerably and several reports have directly compared the various measures as exposure metrics [6,7,10], revealing complex dynamics dependent on several variables. In our initial analysis of risk factors for asthma at age seven [2], we were surprised to find that questionnaire data suggested that maternal smoking was not a significant risk factor and could be a significant “protective” factor. The questionnaire-based data in the current study again suggests “protection” from maternal smoking, though with a less focused confidence interval due to a smaller sample size (in our previous paper [2] biomarkers were not required for inclusion in analysis). As we speculate that maternal smoking is unlikely to be truly protective, these findings motivated our exploration of more objective measurement of exposure, and our findings reinforce that motivation.

Limitations to our data include lack of questionnaire data regarding maternal smoking from the period prior to the 3^rd^ trimester and sample size affecting the confidence limits for the effect of various metrics. This is important because Neuman and colleagues [11] have documented a time-dependency to ETS exposures during early life, though their analysis focused on pregnancy versus post-pregnancy ETS rather than on trimester-specific ETS. Duijts and colleagues [12] did look at trimester-specific effects but for wheeze only (not asthma). Note however that given our specific motivation to compare questionnaire-based data versus that biological sampling, the 3^rd^ trimester would correspond most closely with cord-blood at birth. Another potential limitation is that concern for familial asthma risk led to behavioral changes (decreased smoking) of some family members to reduce the risk of asthma in their children, thus limiting generalizability of our findings in this cohort of children at high risk for asthma, relative to other cohorts with average asthma risk and more typical smoking prevalence. Finally, we did note that amongst those 31 children in the cohort that had both frequent “colds”, defined as at least 3 per year, the risk for wheeze at 2 years (45%) was higher than in those with fewer colds (6% of these children had wheeze at 2 years). Though we were underpowered for a specific analysis of interactions between ETS and upper respiratory tract infections, potential such interactions remain an important concern, as highlighted by Ciprandi and others [13].

In spite of these limitations, our study is unique in evaluating various measures of ETS, in association with multiple respiratory endpoints; we believe that this is further unique in the context of a birth cohort at high risk for asthma but with modest maternal smoking rates. In spite of self-reported maternal smoking occurring in reportedly less than 10% of the families, this metric appears to be the robust for predicting early life recurrent wheeze, and similar risk estimates for this endpoint were noted when ETS was represented by either a broader sampling of family members or by cord blood cotinine. This latter similarity in risk is remarkable since cotinine, while more comprehensively measuring inhaled ETS (including that due to paternal and incidental exposures), has a relatively short half-life of approximately 20 hours [14] and thus may not represent averaged exposure over the specific time period.

## Conclusions

In conclusion, various metrics of ETS exposure focused on the time of birth, but not those focused at 1 year of age, seem to confer increased risk for recurrent wheeze at 2 years, but none of these ETS metrics appear to confer risk for later respiratory outcomes. As recent work by others suggest [15], this pattern may be explained by ETS exposure leading to transient inflammation in the context of underdeveloped 2-year-old airways (precipitating wheezing at age 2), rather than to changes resulting in persistently hyperreactive airways at age 7; ETS exposure may be particularly influential in early-life wheeze, but such influence may be less strong (relative to other exposures that influence respiratory outcomes) by 7 years of life in high risk children. Biomarker- and questionnaire-based assessment of ETS in early life lead to similar estimates of ETS-associated risk of recurrent wheeze and asthma in this high-risk cohort.

## Abbreviations

ETS: Environmental tobacco smoke; CCot: Cotinine in cord blood; UCot: Cotinine in urine; BHR: Bronchial hyperresponsiveness; OR: Odds ratio; R: Pearson correlation coefficient.

## Competing interests

The authors declare that they have no competing interests.

## Authors’ contributions

Authors have made substantial contributions to conception and design (CC, HDW, MCY), acquisition of data (ABB, MCY), analysis and interpretation of data (CC, ADB, ABB, MCY), drafting the manuscript or revising it critically for important intellectual content (CC, HDW, ADB, ABB, MCY), and/or have given final approval of the version to be published (CC, HDW, ADB, ABB, MCY).

## Pre-publication history

The pre-publication history for this paper can be accessed here:

http://www.biomedcentral.com/1471-2431/12/187/prepub

## References

[B1] King ME Mannino DM Holguin F Risk factors for asthma incidence. A review of recent prospective evidence Panminerva Med 2004 46 2 97 110 15507879

[B2] Chan-Yeung M Hegele RG Dimich-Ward H Ferguson A Schulzer M Chan H Early environmental determinants of asthma risk in a high risk cohort Pediatr Allergy Immunol 2008 19 6 482 489 10.1111/j.1399-3038.2007.00689.x 18266835

[B3] Vork K Broadwin RL Blaisdell RJ Developing asthma in childhood from exposure to secondhand tobacco smoke: insights from a meta-regression Environ Health Perspect 2007 115 10 1394 1400 17938726 10.1289/ehp.10155 PMC2022647

[B4] Chan-Yeung M Manfreda J Dimich-Ward H Ferguson A Watson W Becker A A Randomized Controlled Study on the Effectiveness of a Multifaceted Intervention Program in the Primary Prevention of Asthma in High-Risk Infants Arch Pediatr Adolesc Med 2000 154 7 657 663 10.1001/archpedi.154.7.657 10891016

[B5] Daley D Lemire M Akhabir L Chan-Yeung M He JQ McDonald T Sandford A Stefanowicz D Tripp B Zamar D Bosse Y Ferretti V Montpetit A Tessier MC Becker A Kozyrskyj AL Beilby J McCaskie PA Musk B Warrington N James A Laprise C Palmer LJ Pare PD Hudson TJ Analyses of associations with asthma in four asthma population samples from Canada and Australia Hum Genet 2009 125 4 445 459 10.1007/s00439-009-0643-8 19247692

[B6] Puig C Garcia-Algar O Monleon T Pacifici R Zuccaro P Sunyer J Figueroa C Pichini S Vall O A longitudinal study of environmental tobacco smoke exposure in children: Parental self reports versus age dependent biomarkers BMC Public Health 2008 8 1 47 10.1186/1471-2458-8-47 18254964 PMC2276212

[B7] Pichini S Basagana X Pacifici R Garcia O Puig C Vall O Harris J Zuccaro P Segura J Sunyer J Cord serum cotinine as a biomarker of fetal exposure to cigarette smoke at the end of pregnancy Environ Health Perspect 2000 108 1079 1083 10.1289/ehp.001081079 11102300 PMC1240166

[B8] Martinez F Antognoni G Marci F Bonci E Midulla F De Castro G Parental smoking enhances bronchial responsiveness in nine-year old children Am Rev Respir Dis 1988 138 518 523 3202406 10.1164/ajrccm/138.3.518

[B9] Ehrlich R Du Toit D Jordaan E Zwarenstein M Potter P Volmink J Risk factors for childhood asthma and wheezing: importance of maternal and household smoking Am J Respir Crit Care Med 1996 154 681 688 8810605 10.1164/ajrccm.154.3.8810605

[B10] Nafstad P Kongerud J Botten G Urdal P Silsand T Pedersen BS Jaakkola JJK Fetal exposure to tobacco smoke products: A comparison between self-reported maternal smoking and concentrations of cotinine and thiocyanate in cord serum Acta Obstet Gynecol Scand 1996 75 10 902 907 10.3109/00016349609055025 9003090

[B11] Neuman A Hohmann C Orsini N Pershagen G Eller E Kjaer HF Gehring U Granell R Henderson J Heinrich J Lau S Nieuwenhuijsen M Sunyer J Tischer C Torrent M Wahn U Wijga AH Wickman M Keil T Bergstrom A Maternal smoking in pregnancy and asthma in preschool children: a pooled analysis of eight birth cohorts Am J Respir Crit Care Med 2012 186 10 1037 1043 10.1164/rccm.201203-0501OC 22952297

[B12] Duijts L Jaddoe VW van der Valk RJ Henderson JA Hofman A Raat H Steegers EA Moll HA de Jongste JC Fetal exposure to maternal and paternal smoking and the risks of wheezing in preschool children: the Generation R Study Chest 2012 141 4 876 885 10.1378/chest.11-0112 21960695

[B13] Ciprandi G Caimmi D Miraglia Del Giudice M La Rosa M Salpietro C Marseglia GL Recent developments in United airways disease Allergy Asthma Immunol Res 2012 4 4 171 177 10.4168/aair.2012.4.4.171 22754709 PMC3378922

[B14] Knight G Palomaki G Lea D Haddow J Exposure to environmental tobacco smoke measured by cotinine 125I- radioimmunoassay Clin Chem 1989 35 6 1036 1039 2731343

[B15] Sørensen M Allermann L Vogel U Andersen P Jespersgaard C Loft S Raaschou-Nielsen O Polymorphisms in inflammation genes, tobacco smoke and furred pets and wheeze in children Pediatr Allergy Immunol 2009 20 614 623 10.1111/j.1399-3038.2009.00855.x 19674346

